# Optimized Design and Analysis of Sparse-Sampling fMRI Experiments

**DOI:** 10.3389/fnins.2013.00055

**Published:** 2013-04-18

**Authors:** Tyler K. Perrachione, Satrajit S. Ghosh

**Affiliations:** ^1^Department of Brain and Cognitive Sciences, Massachusetts Institute of TechnologyCambridge, MA, USA; ^2^McGovern Institute for Brain Research, Massachusetts Institute of TechnologyCambridge, MA, USA; ^3^Program in Speech and Hearing Biosciences and Technology, Division of Medical Sciences, Harvard Medical SchoolBoston, MA, USA

**Keywords:** sparse-sampling, fMRI, hemodynamic response, auditory neuroscience, HRF, speech perception, speech production

## Abstract

Sparse-sampling is an important methodological advance in functional magnetic resonance imaging (fMRI), in which silent delays are introduced between MR volume acquisitions, allowing for the presentation of auditory stimuli without contamination by acoustic scanner noise and for overt vocal responses without motion-induced artifacts in the functional time series. As such, the sparse-sampling technique has become a mainstay of principled fMRI research into the cognitive and systems neuroscience of speech, language, hearing, and music. Despite being in use for over a decade, there has been little systematic investigation of the acquisition parameters, experimental design considerations, and statistical analysis approaches that bear on the results and interpretation of sparse-sampling fMRI experiments. In this report, we examined how design and analysis choices related to the duration of repetition time (TR) delay (an acquisition parameter), stimulation rate (an experimental design parameter), and model basis function (an analysis parameter) act independently and interactively to affect the neural activation profiles observed in fMRI. First, we conducted a series of computational simulations to explore the parameter space of sparse design and analysis with respect to these variables; second, we validated the results of these simulations in a series of sparse-sampling fMRI experiments. Overall, these experiments suggest the employment of three methodological approaches that can, in many situations, substantially improve the detection of neurophysiological response in sparse fMRI: (1) Sparse analyses should utilize a physiologically informed model that incorporates hemodynamic response convolution to reduce model error. (2) The design of sparse fMRI experiments should maintain a high rate of stimulus presentation to maximize effect size. (3) TR delays of short to intermediate length can be used between acquisitions of sparse-sampled functional image volumes to increase the number of samples and improve statistical power.

## Introduction

The brain bases of speech, hearing, language, and music are increasingly well-understood after two decades of functional neuroimaging research (Price, 2012), despite how inhospitable the magnetic resonance (MR) scanning environment is to the study of these abilities. Acquiring MR images of the entire brain at a timescale conducive to studying physiological function requires rapidly adjusting the scanner’s magnetic gradients, which results in acoustic noise of more than 110 dB (Ravicz et al., 2000). Although the risk to participants’ hearing from such levels is effectively mitigated through passive attenuation, the amount of unattenuated noise remains sufficient to mask auditory stimuli presented at ecological levels. Acoustic noise is rivaled only by participant head motion as the greatest impediment to fMRI studies of speech perception, audition, or music, and head motion poses an additional serious limitation on understanding the brain bases of speech and language, as such motion is inherent to speech production. In an effort to accommodate the MR environment to the study of speech and hearing, no technical advance has had a greater impact on the field than *sparse-sampling* (Scheffler et al., 1998; Edmister et al., 1999; Hall et al., 1999; Talavage and Hall, 2012).

Sparse-sampling is a technique in which a delay is introduced following each functional volume acquisition, during which the scanner gradients are turned off and the MR environment is effectively silent. In this way, sparse-sampling involves acquisition of the same rapid, whole-brain functional volumes available in continuous fMRI, while the silent delays between image acquisitions not only allow auditory stimuli to be presented without the masking effect of the gradients, but also enable participants to produce overt spoken responses without motion-induced noise artifacts in the functional images. Numerous studies have demonstrated that primary and association auditory cortex respond robustly to the acoustic noise generated by the MR scanner. This has been demonstrated by measuring the response to recorded scanner noise (Hall et al., 2000; Gaab et al., 2006b) and by modifying scanner pulse sequences to engage gradient selection without excitation or acquisition during the typically “silent” delay period of sparse-sampling (Hu et al., 2010). The consequence of this functional response to acoustic scanner noise is the “saturation” of physiological signal from auditory cortices, effectively reducing the dynamic range in which a task-evoked neurophysiological response can be measured (Scarff et al., 2004) – an effect that is exacerbated for auditory stimuli sharing frequency components with broadband scanner noise (Langers et al., 2004).

A number of variations on sparse-sampling’s core idea of inserting silent delays between acquisitions have been proposed over the years. In the original introduction of sparse-sampling, silent delays were used to accompany classic block-designs for stimulus presentation (Scheffler et al., 1998; Hall et al., 1999). Sparse-sampling has also been used to facilitate event-related fMRI, enabling researchers to determine the evoked hemodynamic response function for auditory stimuli (Belin et al., 1999). This classical approach to event-related sparse fMRI is time-consuming – only one stimulus can be presented per volume acquisition (TR) – and does not take advantage of statistical improvements giving rise to “rapid” event-related fMRI (Dale, 1999). [The implications for sparse-sampling on other classical experimental design approaches are considered by Amaro et al. (2002)]. Another early description of sparse-sampling termed this technique “clustered volume acquisition” (Edmister et al., 1999), because all slice acquisitions occurred in a “cluster” at the beginning of each TR, rather than distributed evenly throughout the TR as is usual for continuous imaging. This choice of terminology has resulted in an unfortunate methodological ambiguity in the field, given a later permutation of sparse-sampling involving the collection of multiple functional volumes subsequent to a single silent delay, which has been similarly termed “clustered temporal acquisition” (Bandettini et al., 1998; Zaehle et al., 2007)^1^. A major limitation of clustered temporal acquisition for sparse designs is that temporally adjacent volumes will differ in T1 contrast due to a failure to reach a stable longitudinal magnetization. Schwarzbauer et al. (2006) developed a refinement of clustered temporal acquisition to address this problem called “interleaved silent steady state” (ISSS) imaging, in which slice excitation is maintained even during silent delay periods, allowing stable longitudinal magnetization to be achieved and image contrast to remain consistent across adjacent volumes. However, the ISSS technique does not appear to be in wide use (see Current Practices in Sparse fMRI Design and Analysis, below).

The effectiveness of sparse-sampling in improving the detectability of auditory-evoked neurophysiological response in fMRI has been repeatedly demonstrated. Compared to continuous acquisition, increased response in auditory cortices has been found for sparse-sampling with both short and long silent delays between acquisitions (Gaab et al., 2006a; Blackman and Hall, 2011), and a more extensive cortical network related to speech comprehension is observed in studies using sparse than continuous sampling (Adank, 2012). Similar improvements have been found for clustered sparse acquisitions versus continuous ones (Schmidt et al., 2008). In addition to research on humans, sparse-sampling has been shown to offer compelling improvements over continuous imaging for auditory fMRI in non-human primates (Petkov et al., 2009). Additionally, sparse-sampling has been able to reveal the effects of acoustic noise on neurophysiological brain activity unrelated to auditory tasks. Acoustic scanner noise suppresses the default-mode network, suggesting that such noise impairs the brain’s ability to reach a true “resting state” (Gaab et al., 2008; Langers and van Dijk, 2011). Differential cortical activation in frontal and parietal areas during a visual working memory task reveals that continuous acoustic scanner noise has a significant effect on even non-auditory attentional and working memory processes (Tomasi et al., 2005). However, sparse-sampling is not without its drawbacks: if volume acquisition time (TA) is 2.0 s, then in a sparse-sampling experiment employing a 12 s TR, a 5 min functional run will collect only 25 volumes, compared to the 150 volumes that would have been collected during continuous scanning. This difference in sample size is a non-trivial detriment to the power of sparse-sampling fMRI studies and can result in Type II errors for experimental effects, which may manifest as reduced areal activation (Nebel et al., 2005).

In addition to sparse-sampling, other attempts have been made at reducing the functional consequences of acoustic noise during auditory fMRI, including passive attenuation of noise via ear plugs, ear muffs, and even whole head-encompassing helmets (Ravicz and Melcher, 2001); imaging sequences specially designed to reduce gradient-related acoustic noise (Seifritz et al., 2005; Peelle et al., 2010); and even active attenuation of acoustic noise with MRI-compatible noise-canceling headphones (Hall et al., 2009; Blackman and Hall, 2011). Of all these techniques, sparse-sampling remains the most effective and least technically convoluted way of reducing the effect of acoustic scanner noise. Moreover, despite offering limited improvement for auditory fMRI, none of these other noise-attenuation techniques can simultaneously reduce the impact of head motion-induced noise associated with speech production (although other approaches have been suggested: Birn et al., 2004; Gracco et al., 2005).

### Current practices in sparse fMRI design and analysis

Given limited prior work exploring the parameterization of sparse design and analysis techniques, we were curious to see when and how sparse-sampling fMRI was currently being used to investigate the neural bases of speech and audition. We conducted a query of the Thompson Reuter’s *Web of Science* database for all papers published in the year 2011 matching the following terms in the Topic field: (“auditory” or “speech” or “spoken language” or “voice” or “vocal” or “music” or “sound” or “acoustic”) and (“fMRI” or “functional MR” or “functional MRI”). We limited the search to indexed journals that have been principal outlets for speech/auditory fMRI studies, resulting in 168 papers across the following journals (N): *Brain* (4), *Brain and Language* (11), *Cerebral Cortex* (16), *Human Brain Mapping* (17), *Journal of Cognitive Neuroscience* (31), *Journal of Neuroscience* (20), *Nature Neuroscience* (2), *NeuroImage* (64), and *Neuron* (3). (The references for these articles, and their classification in our survey, are available in this project’s online supplementary materials). Of these 168 papers, 146 reported novel fMRI experiments (as opposed to meta-analyses, tests of new methods on previously published data, or reporting no fMRI experiment), of which 107 used audio stimuli, 13 involved overt speech production, and 6 involved both speech production and audio stimuli.

**Figure 1:**
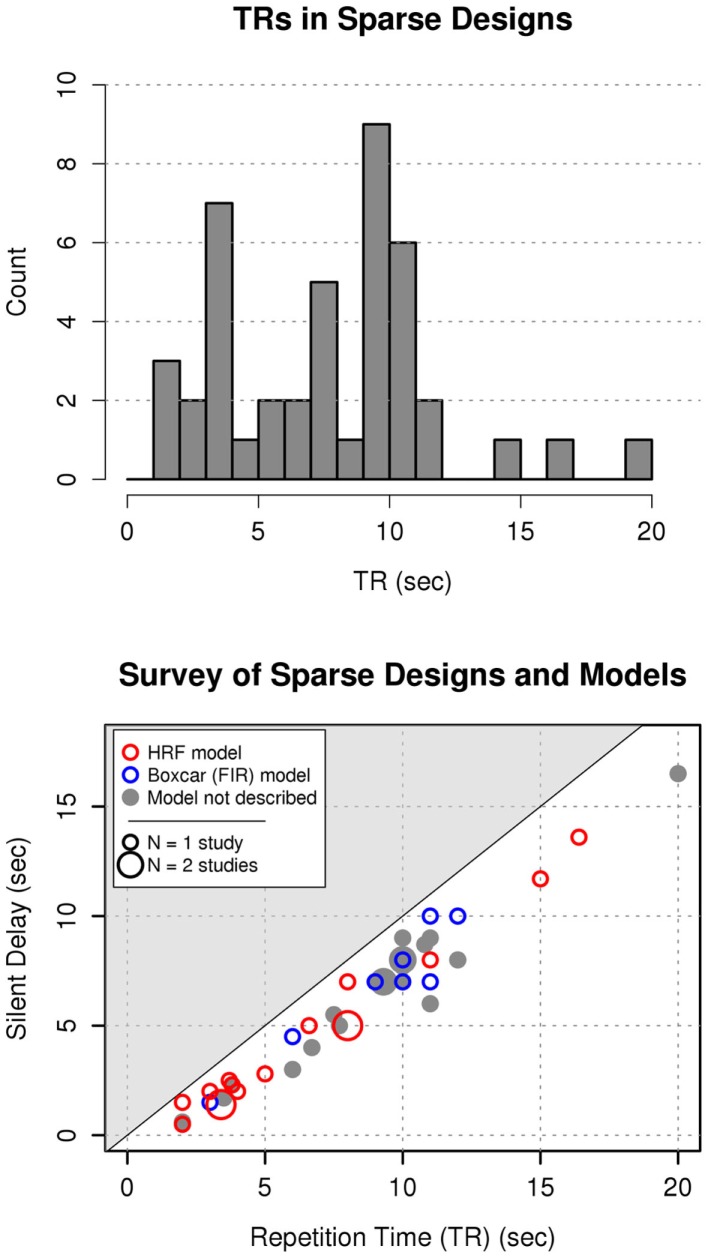
**Survey of contemporary sparse-sampling fMRI design and analysis methods**. Top panel: of papers published in 2011, two principal modes of sparse design were observed – those involving short TRs (≤4 s), and those involving long TRs (9–12 s), with comparatively fewer using intermediate values. Bottom panel: choice of sparse analysis model across designs. Red outlined circles = Models employing HRF convolution; Blue outlined circles = Boxcar (FIR) models; Filled gray circles = studies with no description of the model basis functions. Smaller circles are *N* = 1 study at that point, larger circles are *N* = 2 studies. Continuous sampling studies would lie on the line *y* = *x*. The silent delay cannot exceed the TR (shaded region; TR = TA + silent delay).

Of the 114 papers investigating speech or audition, 43 used some variant of a sparse-sampling technique (8 of 13 speech papers; 40 of 107 auditory papers). A wide range of sparse design choices were attested: repetition times (TR) ranged from 2.0 to 20.0 s; silent delays from 0.5 to 16.5 s; and acquisition times (TA) from 0.5 to 3.5 s. A majority of studies adopted either of two approaches to sparse design (Figure 1): short TRs (<5 s) with short silent delays (<3 s), or long TRs (9–12 s) with long silent delays (5–10 s). The majority of sparse designs involved the acquisition of a single functional volume followed by a delay in which the gradient magnetic fields were turned off and no signal was acquired (*N* = 38). Three papers utilized clustered temporal acquisition, in which multiple volumes (2–5) were acquired consecutively between each silent delay, of which two papers reported the use of the ISSS technique (Schwarzbauer et al., 2006). There was considerable heterogeneity in the reported analysis techniques for sparse designs, with 8 papers reporting the use of FIR (boxcar) model designs, 16 papers reporting model designs incorporating HRF convolution, and 19 papers not reporting their models’ basis functions (Figure 1, bottom panel). None of the papers incorporating hemodynamic response convolution explained how the design matrix accounted for temporal discontinuity in the measured signal.

### The present study

**Figure 2:**
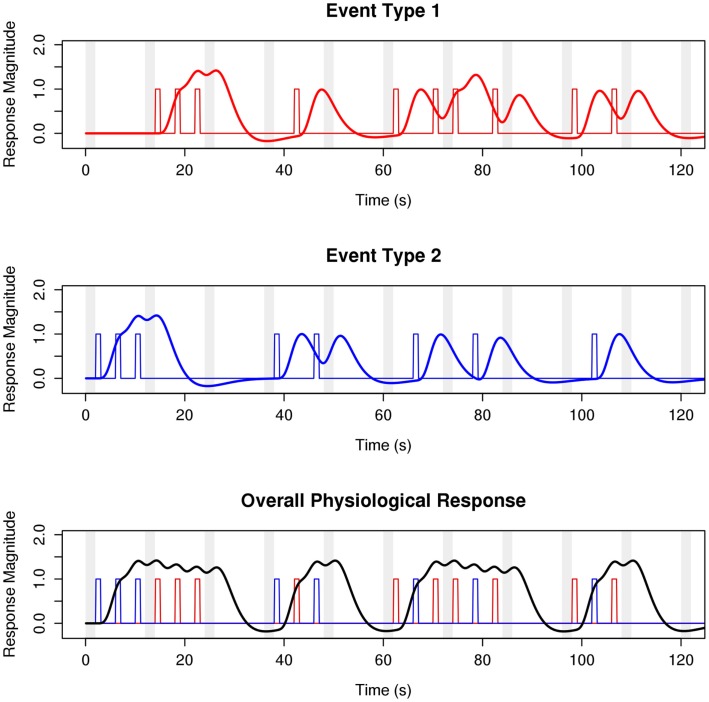
**Sparse-sampling captures only fractions of (overlapping) hemodynamic responses to stimulus events**. A simulated experimental design with two event types is shown. Stimuli are presented during the silent delay between acquisitions. Events of both types occur during the delay period of a single TR, such that the MR signal sampled during the TA contains information about both event types. Classical approaches to sparse analysis are unable to disentangle signal from different event types, consequently limiting sparse experiments block or slow event-related designs. The HRF-convolution approach we advocate in this paper allows for rapid event-related sparse-sampling fMRI experiments. Legend: red/blue distinguish event types; colored vertical bars denote stimulation events; red and blue curves show the canonical hemodynamic response to these events; the black curve shows the aggregate response across event types; vertical gray bars indicate time of MR signal acquisition; TR = 12 s; TA = 2 s; ISI = 4 s.

Despite the considerable benefit afforded by the sparse-sampling technique, and despite its ubiquitous use in auditory cognitive neuroscience over the last decade, there has been little systematic investigation of the optimal acquisition parameters, experimental design considerations, and statistical analysis approaches that bear on the results and interpretation of sparse-sampling fMRI experiments. Although classic sparse designs call for very long TRs with long silent delays (which allow the physiological response to scanner noise to return to baseline before the next volume is sampled), a recent report that tested delays between 7.5 and 15.0 s during clustered temporal acquisition found that shorter delays may be advantageous (Liem et al., 2012). As surveyed above, contemporary studies are using silent delays anywhere between 0.5 and 16.5 s, although quantitative work has not previously examined the relative efficacy of delays in this range. Similarly, classical event-related sparse designs call for a single stimulus to be played per silent delay to facilitate mapping the hemodynamic response (Belin et al., 1999). However, slow event-related designs do not make optimal use of expensive scanner time, and statistical techniques have been known for some time that allow for rapid event-related fMRI (Dale, 1999). Employing rapid event-related designs for sparse fMRI requires a change in how sparse-sampled datasets are analyzed: current analysis practices typically utilize a boxcar model design, especially for acquisitions with long silent delays. However, boxcar model designs are unable to distinguish between overlapping responses to different stimulus types measured during a single volume acquisition (Figure 2). To ameliorate this problem, and to facilitate the use of rapid event-related designs in sparse-sampling fMRI, the efficacy of models incorporating information about the physiological response (HRF) must also be assessed.

In this report, we examine how design and analysis choices related to the duration of the sparse delay, rate of stimulus presentation, and model basis functions act independently and interactively to affect the neural activation profiles observed in fMRI. First, we conducted a series of computational simulations to explore the parameter space of sparse design and analysis with respect to these variables. Second, we validated the results of these simulations in a series of sparse-sampling fMRI experiments. Following these results, we offer a set of suggestions for the design and analysis of sparse-sampling fMRI experiments that will allow researchers to take advantage of these optimizations when they might be appropriate.

## Computational Exploration of Sparse-Sampling fMRI Design and Analysis Parameter Space

We developed a computational simulation of the fMRI time series of an auditory experiment in order to explore the parameter space of sparse design and analysis. In particular, we were interested in the effects of sparse delay (effective acquisition rate, TR) and stimulation rate (inter-stimulus interval, ISI) as design parameters, and the effects of a physiologically informed model (including convolution of the canonical HRF) as an analysis parameter. By developing a computational simulation of the effects of, and interactions among, these parameters, we sought to identify possible optimizations on current sparse designs without the time and expense of exploring their full parameterization in the scanner with actual human subjects. Subsequently, we sought to validate the results of these simulations through comparative analysis of real fMRI data.

### Methods

Simulations were conducted in **R** (version 2.15.2)^2^ on a Dell Optiplex 760 running Fedora Linux 17 (kernel 3.7.9-104.fc17.x86_64). Multiple iterations of the simulation were conducted, and the results of the models (parameter estimates, *t*-statistics, and residuals) were recorded to determine how the mean and range of these values varied according to simulation parameters. Each iteration of the simulation can be thought of as a separate fMRI session, and the observed differences in design or analysis choices can be thought of as differences in the results of first-level (within-subject) fMRI results. The parameters that were varied across iterations of the simulations included the length of the sparse delay (effective TR), stimulation rate (ISI), the times of event onsets, and the temporal noise profile. The parameters that were varied within a single iteration of the simulation included the temporal signal-to-noise ratio (tSNR) and model type (HRF or boxcar). The parameters that were held constant across iterations included event duration (1.0 s), acquisition time (TA, 2.0 s), delay between the onset of an event, and the offset of the previous TA (0.5 s), canonical HRF shape, proportion of “rest” TRs (33%), temporal resolution of the simulated time series (100 ms), and the total run length (360 s)^3^.

**Figure 3:**
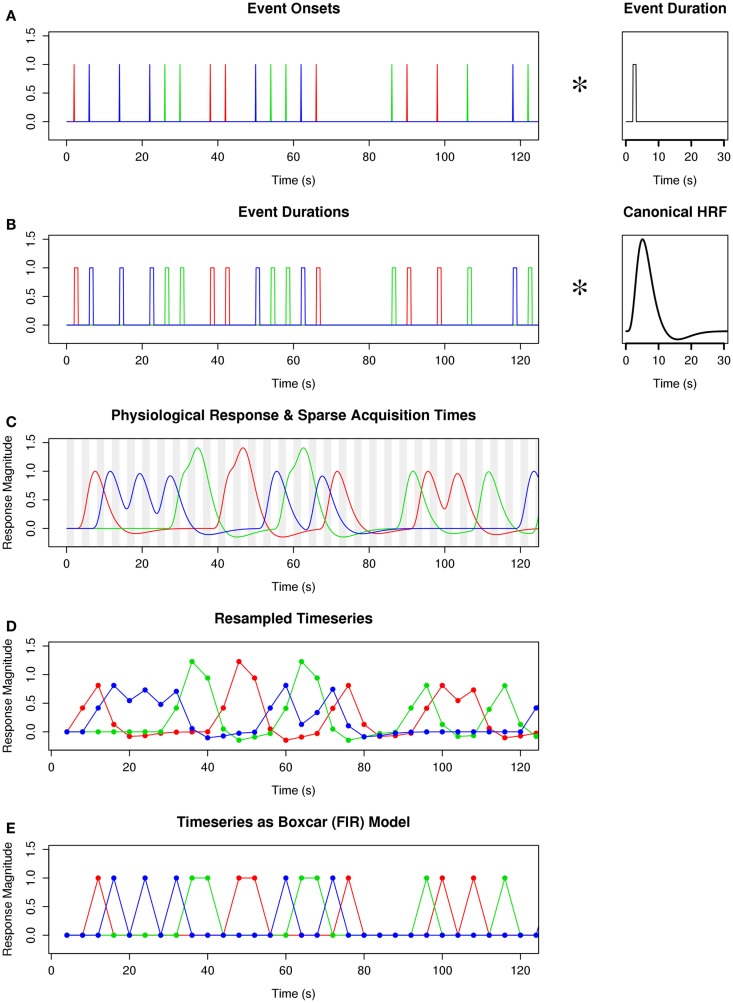
**Procedure for convolving HRF with sparse-sampled time series**. **(A)** A high temporal resolution vector of event onsets is convolved with a function of their durations. **(B)** The resulting time series is convolved with the canonical hemodynamic response function. **(C)** The ideal physiological response is resampled (without filtering) at the time points when actual MR signal was acquired. **(D)** The resulting resampled time series can be scaled and demeaned for use in the design matrix of the general linear model. **(E)** The same sparse time series is shown with classical boxcar (FIR) modeling, illustrating the greater information in the HRF-convolved time series. Figure legend: colored traces (red, green, blue) depict different event types; vertical gray bars indicate time of MR signal acquisition; TR = 4 s; TA = 2 s; ISI = 4 s.

On each iteration, a list of random event onset times was generated, such that event onsets were separated by the ISI, the first event of a TR began after a fixed delay, the full duration of each event occurred only during the simulated silent portion of each TR, and rest trials lasted the full duration of a TR (because of the limitations of the boxcar model, as shown in Figure 2). The list of event onsets was converted into a high-temporal resolution vector of delta functions occurring at event onsets (Figure 3A). Each delta function was convolved with a square wave whose width equaled the duration of the event associated with the delta function, resulting in a stimulation timeline (Figure 3B). The stimulation timeline was convolved with a canonical HRF to produce a high-temporal resolution vector of simulated physiological response to stimulation (Figure 3C). (The amplitude of the canonical HRF used in this convolution was scaled so that the peak of this HRF when convolved with a solitary 1.0 s event would equal 1.0, corresponding to a canonical percent signal change of 1% for a 1 s stimulation). The mean value of the high-temporal resolution response vector was computed over each time window corresponding to the TAs when “actual” scanner data would be acquired, and the resulting values were concatenated into a resampled time series whose length equaled the number of TAs (Figure 3D). A corresponding “boxcar” vector was generated such that every TA in which an event had occurred was encoded with a “1,” and every TA in which no events had occurred was encoded with a “0” (Figure 3E). (For TRs of 4 s or less, the TA associated with an event was delayed by one TR to better simulate capturing the peak of the hemodynamic response). The resampled response time series was de-meaned and scaled so that its peak-to-peak height equaled 1.0, insuring that a parameter estimate of 1.0 would signify a 1% percent signal change in physiological response. The resampled and scaled HRF response vector is designated *x*_hrf_. The boxcar vector, already having a peak-to-peak height of 1.0, was only de-meaned; this response vector is designated *x*_box_.

**Figure 4:**
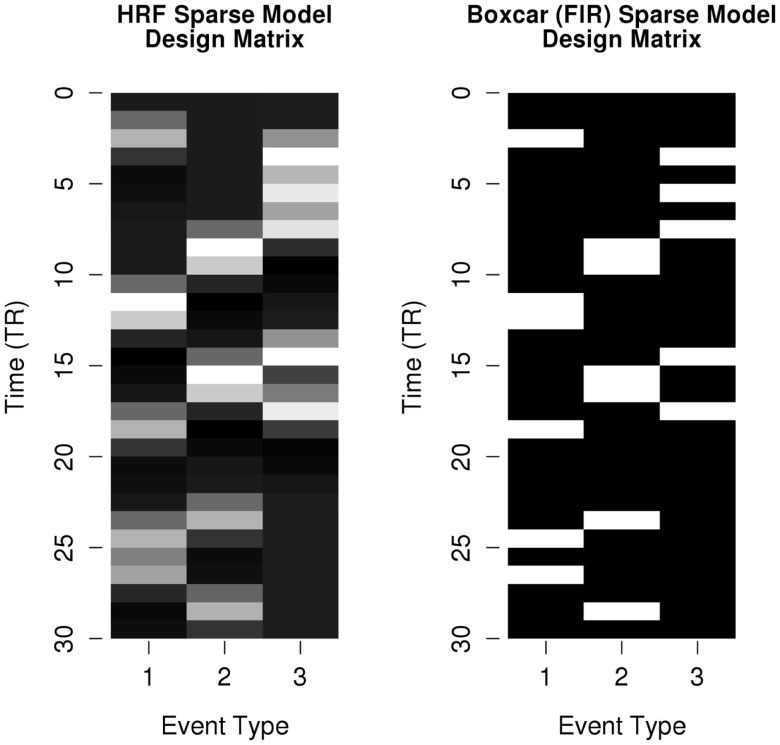
**Example design matrices for convolved-HRF or classic boxcar (FIR) sparse models**. Design matrices modeling event types of the simulated time series shown in Figure 2. Design matrix parameters that do not differ between models (overall mean, nuisance variables, etc.) are not shown.

The resampled HRF time series was considered the “ideal physiological response” and was used as the basis for the response vector *y* in the linear models. To simulate the measurement noise associated with fMRI data, a random vector of Gaussian noise (μ = 0; σ = 1) was generated. The Gaussian distribution has been shown to effectively capture physiological noise in fMRI time series (Wink and Roerdink, 2006).The root-mean-square (RMS) amplitude of the noise vector was scaled so that the tSNR of the combined ideal response plus noise vector was of a fixed value: RMS(*A*_Noise_) = RMS(*A*_Signal_) ÷ 10^tSNR÷20^. The scaled noise vector was then added to the resampled HRF time series to produce the simulated fMRI time series *y*. For each time series design and noise profile, we estimated models with tSNR values between −20 and 10 dB (in integer steps) to simulate the range of potential noise profiles found across experiment designs and scanner hardware. The linear models *y* = β*x* + ε were then estimated separately for both *x*_hrf_ and *x*_box_ (see Figure 4 for a comparison of these design matrices), and the parameter estimate, *t*-statistic of the parameter estimate, and residuals for each model were recorded for comparison across the various permutations of simulation parameters. (The full simulation code is available in this project’s supplementary materials online.)

Following, we report the results of simulations of the following sparse delay × stimulation rate permutations (TR × ISI), in seconds: 4 × 4, 8 × 4, 12 × 4, 4 × 8, 8 × 8, 3 × 3, 6 × 3, 9 × 3, 6 × 6, 9 × 9. The results describe the values obtained after 100 simulations of each TR × ISI permutation.

### Results

#### Differences between HRF and Boxcar models

**Figure 5:**
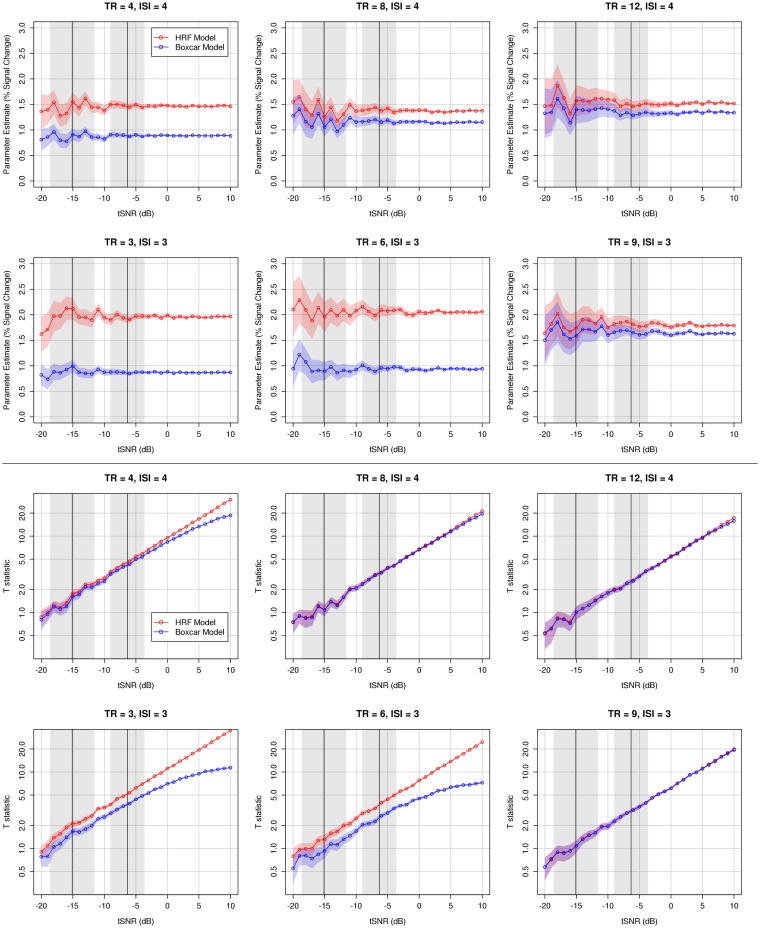
**Comparison of HRF and boxcar models from simulated designs for various acquisition (TR) and stimulation (ISI) rates**. Red traces illustrate mean values for the HRF model; blue traces, boxcar model. Colored shaded regions accompanying traces indicate the 95% confidence interval of the mean over 100 simulations (see text). The accuracy and precision of the parameter estimate improves with increasing tSNR. Classical boxcar models are likely to substantially underestimate the effect size (percent signal change) of a stimulus at all TR lengths. For shorter TRs, the HRF model results in a substantially more statistically reliable model than a classic boxcar model, an effect that is enhanced with increasing tSNR. The range of actual tSNR values observed in the experimental validation of these results are illustrated in the shaded gray domains: left domain, mean and 95% confidence interval across participants of the *mean* tSNR within left Heschl’s gyrus; right domain, mean and 95% confidence interval across participants of the *maximum* tSNR in left Heschl’s gyrus. Note that the plots showing *t*-statistics are effectively log–log plots.

Compared to the HRF model, the classic boxcar model consistently underestimated the magnitude (percent signal change) of the simulated physiological response, as encoded in the model parameter estimate (Figure 5, upper panels). This difference was especially pronounced for short TRs (<8 s), where the effect size reported by the boxcar model could underestimate the true response by a factor of 2. Even for longer TRs (≥8 s), when the simulated time series had moderate (≥−10 dB) or high tSNR, the boxcar model consistently underestimated the response magnitude. For both models, the variability associated with the parameter estimate across simulations decreased as a function of tSNR, such that high signal-to-noise ratio allowed more consistent response estimation.

**Figure 6:**
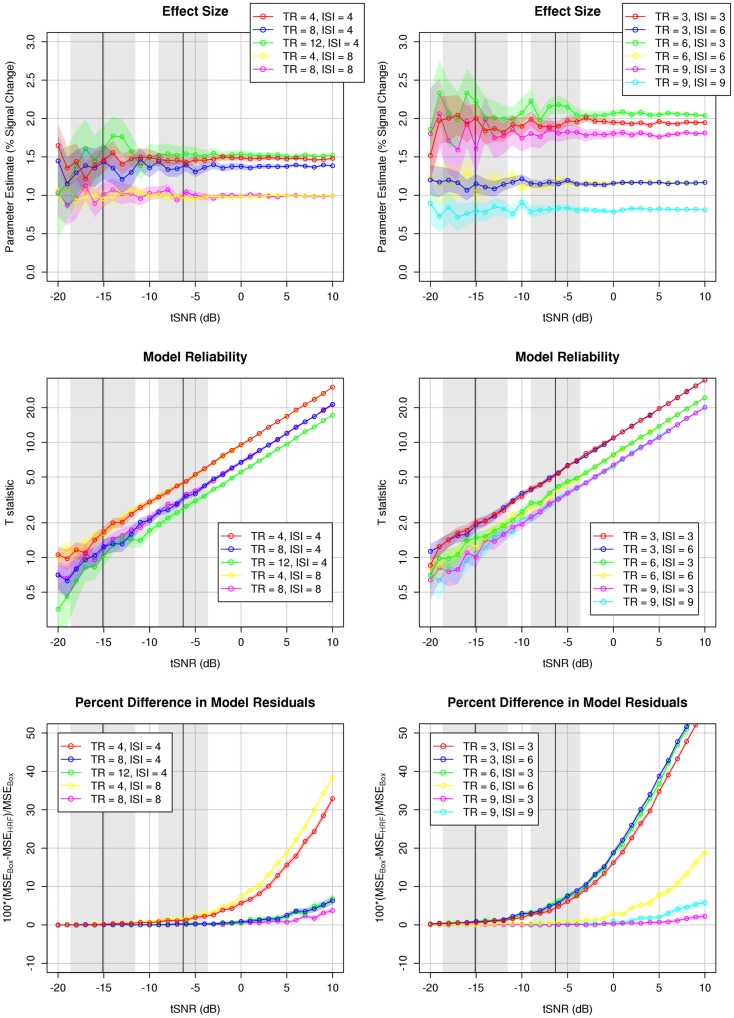
**Comparison of acquisition (TR) and stimulation (ISI) rates**. Colored traces indicate different TR/ISI permutations; colored shading depicts the 95% confidence interval over 100 simulations (see text). More frequent stimulation designs result in larger parameter estimates (percent signal change) due to additivity of the BOLD response. Shorter TRs afford increased model reliability compared to longer TRs. Stimulation and acquisition rates interact in affecting the relative benefit of the HRF model (as assessed by lower model residuals), such that the HRF model results in less error for short TRs with rapid stimulation, and long TRs with infrequent stimulation, again likely due to the additivity of the BOLD response. The range of actual tSNR values observed in the experimental validation of these results is illustrated in the shaded gray domains: left domain, mean and 95% confidence interval across participants of the *mean* tSNR within left Heschl’s gyrus; right domain, mean, and 95% confidence interval across participants of the *maximum* tSNR in left Heschl’s gyrus. Note that the plots showing model reliability are effectively log–log plots.

In addition to improved estimation of effect size and increased statistical reliability, sparse models incorporating the hemodynamic response were also associated with reduced model residuals (Figure 6). Acquisition rate and stimulation rate made interacting contributions to the difference in residuals between models. In general, more frequent acquisition rates were associated with smaller residuals in HRF models, and this difference was generally, though not always, emphasized by slower stimulation rates. Overall, more frequent acquisitions made a bigger difference between models than differences in stimulation rate, with shorter acquisitions resulting in substantially smaller residuals when estimated by HRF models.

#### Effects of acquisition rate and stimulation rate on HRF models

Given the marked, consistent improvement in time series modeling when incorporating the physiological response, we ran simulations to investigate how varying the acquisition rate and stimulation rate affected the estimate and reliability of HRF models (Figure 6). More frequent stimulation was associated with larger parameter estimates (i.e., larger effect size, larger percent signal change) independent of acquisition rate – likely the result of additivity of overlapping BOLD responses to subsequent stimulus events (Dale and Buckner, 1997). Acquisition rate had modest, if any, effect on the magnitude of the parameter estimate, and these non-linear differences were likely the result of sampling different regions of the hemodynamic response.

The reliability of HRF models, as measured by the *t*-statistic on the parameter estimate, varied principally as a function of acquisition rate, such that a more rapid acquisition rate was associated with greater model reliability. This difference in model reliability as a function of acquisition rate is likely due primarily to a reduction in standard error as a function of increased sample size, reducing the denominator of the test statistic in the context of a constant numerator (given the observation of relative independence between acquisition rate and measured effect size).

### Discussion

The results of the simulations suggest three principal optimizations of sparse-sampling fMRI data acquisition and analysis: (1) More frequent stimulus presentation (shorter ISIs) increases physiological response and thereby effect size; (2) more frequent acquisition (shorter TRs, less TR delay) reduces error related to measurement noise and increases model reliability; and (3) a physiologically informed model incorporating the canonical hemodynamic response not only provides a more accurate measure of effect size (percent signal change), but also affords greater model reliability and therefore statistical significance, than classical sparse modeling via boxcar design.

The notion that more frequent stimulation is desirable for maximizing BOLD effects is not new to these results: sparse-sampling fMRI experiments have often been designed with numerous stimulation events during a long (8–12 s) delay in TR. This is in contrast to slow, “event-related” sparse imaging, in which a single event is presented during a TR, with jittered presentation time with respect to the TA to allow deconvolution of the shape of the hemodynamic response (Belin et al., 1999). These simulations do suggest, however, that the benefit from more frequent stimulation is not fully realized when the TR is very long, as in classic sparse designs, and when the modeled design does not take into account physiological realities such as the hemodynamic response.

Perhaps the greatest departure of the recommended optimizations from classic sparse designs is the notion that shorter TRs are actually more desirable because of the increased statistical reliability they afford. In classic sparse designs, very long TR delays are used not only to allow auditory stimuli to be presented during silence, but also to allow the hemodynamic response to acoustic noise associated with image acquisition to return to baseline prior to acquiring the next image. Although such an approach appears warranted for reducing contamination of the task-evoked response, it may ultimately be counter-productive because having fewer sample volumes results in greater model error, and therefore both reduced model reliability (*t*) and reduced statistical significance (*p*). Instead, the simulated results suggest that maximal statistical reliability can be achieved by increasing the acquisition rate. However, because the simulations were unable to capture physiological non-linearities leading to saturation or attenuation of task-evoked response magnitude as a function of acoustic noise (with increasing sampling rate), this conclusion in particular demands verification from experiments acquiring actual fMRI data (see below).

Reducing the TR not only allows for the collection of more volumes and the reduction of model error, it also has the added benefit of increasing the model’s degrees of freedom, thereby increasing its statistical significance. That is, in a given scanning session of fixed duration, a sparse design with longer TRs will not only have reduced *t*-statistics due to greater model error, each *t*-statistic value will be associated with lower statistical significance than it would with shorter TRs due to reduced degrees of freedom in the long TR design. Even small differences in the *p*-value of a given voxel are non-trivial in light of current practices in fMRI analysis, where multiple comparisons corrections and activation map thresholds are based on arbitrary *p*-values, and Type II error for individual voxels may thus contribute to Type II error for entire clusters.

A final suggestion based on the simulations is the importance of constructing physiologically informed models for design matrices, even for analysis of sparse-sampling fMRI experiments. Compared to classical boxcar models, models that incorporated a convolved hemodynamic response afforded enhanced statistical reliability across all tSNRs. For moderate tSNR values, differences in the *t-*statistic between models often easily represented an order of magnitude difference in statistical significance – an important discrepancy given the consequences of arbitrary *p*-value cutoffs in fMRI analysis. Additionally, the benefit of the HRF models was most apparent for shorter TRs (namely, those <8 s), which is particularly important given the added benefit of short TRs for improving statistical reliability. Experiments seeking to improve their statistical ability to detect signal by using shorter TRs are exactly those that will additionally benefit most from the use of a physiologically informed approach to model design.

In addition to the increased statistical reliability of a physiologically informed model, there were marked differences in the magnitude of the parameter estimates of the HRF and boxcar models, such that the boxcar model sometimes drastically underestimated the stimulation-related effect size. This difference is likely due to the unique ability of the HRF model to capture two known properties of the hemodynamic response: scaling (increased response with increased stimulus magnitude) and additivity (summation of response to multiple stimuli). Although the height of the boxcar model is fixed for every sampling point regardless of the dynamics of the actual physiological response at that point (Figure 3E), the height of the HRF model can encode differences in the sample-by-sample expected magnitudes (Figures 3C,D). This difference suggests that, in analyses of fMRI experiments modeled with boxcar design, reports of percent signal change based on model parameter estimates may drastically underestimate the true magnitude of the physiological effect.

### Factors potentially limiting the effectiveness of these approaches

In real-world scanning situations there are a number of additional factors that may diminish the ability of the three optimizations suggested by the simulations to facilitate detection of task-related fMRI effects: there may be differential deviation across subjects with respect to the canonical hemodynamic response (Aguirre et al., 1998), and the shape of the hemodynamic response may vary depending on stimulation factors (Harms and Melcher, 2003), potentially reducing the efficacy of the HRF model. Acoustic noise related to image acquisition may contribute to saturation of auditory cortex response and reduce the dynamic range available for detection of stimulus-elicited BOLD signal (Langers et al., 2004; Scarff et al., 2004), potentially reducing the statistical benefit from more frequent acquisitions. Shorter TRs reduce the contrast between tissue types in T2-weighted imaging, potentially affecting the accuracy of fMRI data preprocessing algorithms that depend on tissue contrast, such as motion correction and anatomical coregistration, and reducing the statistical benefit from shorter TRs. Although the hemodynamic response is conventionally treated as fully linear with respect to scaling and additivity, there are known non-linearities, such as physiological adaptation to stimulus repetition (Grill-Spector and Malach, 2001), that may potentially reduce both the accuracy of the HRF model and the effect size benefits of more frequent stimulation. Finally, there may also be unpredictable differences relating to psychological state under short versus long delays between acquisitions (Tomasi et al., 2005; Gaab et al., 2008), or differential physiological response properties across brain regions (e.g., sensory versus frontal cortices) that may detract from (or contribute to) the relative benefit of any of the three optimization techniques. As such, we sought to validate these optimization techniques by assessing, in actual fMRI experiments, experimental designs paralleling those from the simulations.

## Validation of Computational Modeling from Actual fMRI Data

### Methods

To evaluate choices related to the design and analysis of sparse-sampling fMRI experiments, we scanned participants under three different TR delays and with two different stimulation rates while they performed a challenging Stroop task. We wanted to assess these design and analysis choices across brain areas involved in perception, cognition, and action. As such, the Stroop task involved both auditory and visual stimulation, linguistic processing, motor output, and a challenging cognitive go vs. no-go decision on each trial, and it was expected to drive activation in an extensive neural network associated with these functions.

#### Subjects

Healthy adult control participants (*N* = 13), age 21–31 years (*M* = 25) gave informed, written consent overseen by the MIT Committee on the Use of Humans as Experimental Subjects to participate in this study. Participants self-reported being right-handed native English speakers, free from neurological, psychological, visual, or hearing impairments. One additional participant was recruited for this study but excluded from analysis for not completing all experimental conditions.

#### Stimuli

Auditory stimuli consisted of the color words “red,” “green,” “blue,” and “white,” spoken by a female native English-speaking adult and digitally recorded using the software Praat^4^ via a Roland UA-25EX sound card sampling at 44.1 kHz. Auditory stimuli were spectrally filtered to attain frequency response equalization for binaural presentation via a pair of Sensimetrics (Malden, MA, USA) S-14 MRI-compatible insert earphones at a comfortable level for each subject (∼70 dB SPL). Visual stimuli consisted of the color words “red,” “green,” “blue,” and “white,” written in lower-case bold Arial font in either red, green, blue, or white font color (permuted) at full saturation on a dark gray background of ∼20% luminance. Visual stimuli were projected onto a screen at the end of the scanner bore and viewed via a head coil-mounted mirror.

#### Procedure

In the scanner, participants underwent a go/no-go variant of the Stroop task designed to drive response in auditory, visual, cognitive, and somatomotor regions. In this task, one of the four color words was presented in one of the four font colors. Visual stimuli remained on the screen for 750 ms. Simultaneous to the onset of the visual stimulus, one of the four audio stimuli was also presented. Participants were instructed to respond immediately by button press when the color word they heard matched the color of the text (“go” trials), and not to respond when the color word they heard matched the content of the text or when the content of the text matched the color of the font (“no-go” trials). Half of the stimulus events were “go” trials. All participants indicated the task was challenging and maintained their attention throughout the scanning session.

**Figure 7:**
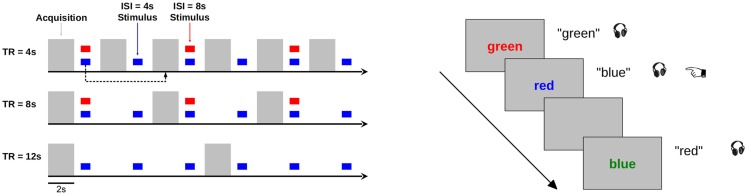
**Design of validation fMRI experiment**. Neurophysiological responses to two stimulation rates (ISI = 4 and 8 s) were measured during three acquisition rates (TR = 4, 8, and 12 s). Gray bars depict acquisition times; colored boxes depict stimulation events. For boxcar models, the event was modeled at the acquisition ~6 s after stimulation in order to capture the peak of the hemodynamic response. For the TR = 8 and 12 s acquisitions, this was the TA immediately following the event; for TR = 4 s acquisition, this was the subsequent TA (as indicated by the black dashed arrow). The task design is illustrated at right. In a modified Stroop task, a color word was displayed in a colored font. Concurrently, participants heard the name of a color spoken over the headphones. Participants pressed a button (illustrated by the pointing finger) whenever the spoken color word matched the text color.

This task was used across five experimental conditions, in which we parametrically varied the sparse delay (repetition time, TR) and stimulation frequency (inter-stimulus interval, ISI). Sparse delays were 2, 6, or 10 s (TR = 4, 8, 12 s, respectively), and stimulation frequency was every 4 or 8 s. Participants completed two runs of each TR × ISI permutation, except for TR = 12 s, ISI = 8 s, which was not included in this study because stimulation would not have occurred consistently with respect to acquisition in every TR. In all conditions, the first stimulus of a TR occurred 500 ms following the previous image acquisition to avoid forward masking as a result of acquisition acoustic noise. The various time series designs are illustrated in Figure 7. For runs with ISI = 4 s, 60 stimuli were presented; for runs with ISI = 8 s, 30 stimuli were presented. One third of the TRs in each run were rest, during which no stimuli were presented and the screen remained dark gray. No stimuli were presented during slice excitation and image acquisition. The task lasted 360 s for each functional run.

#### fMRI data acquisition

Data were acquired on a Siemens Trio 3T scanner with a 32-channel phased array head coil in a single imaging session. A whole head, high-resolution T1-weighted, magnetization-prepared rapid gradient echo (MPRAGE) anatomical volume (acquisition parameters: TR = 2350 ms, TE = 1.79 ms, flip angle = 7°, TI = 1400 ms, voxel resolution = 1.0 mm × 1.0 mm × 1.0 mm, FOV = 256 × 256, 176 sagittal slices) was collected prior to the functional runs.

Two functional runs were collected in each of the five acquisition/stimulation rate conditions using sparse-sampled T2^*^-weighted gradient echo echo-planar imaging (EPI) scans [acquisition parameters: TR = (4.0/8.0/12.0 s), TA = 2.0 s, delay = (2.0, 6.0, 10.0 s), TE = 30 ms, flip angle = 90°, voxel resolution = 3.125 mm × 3.125 mm × 4.0 mm, FOV = 64 × 64, 32 transverse slices acquired parallel to the AC-PC plane, providing whole-brain coverage, number of volumes = (92, 47, 32)]. Each functional run was preceded by three additional TRs during which no data were recorded to allow for stabilization of longitudinal magnetization. (The full details of the MR acquisition parameters are available in this project’s supplementary materials online.)

#### fMRI data analysis

Preprocessing, analysis, and presentation of fMRI data reported here were achieved using the following software packages: Nipype v0.5 (Gorgolewski et al., 2011)^5^ and standard processing pipelines from BIPs^6^, FSL v4.1.6 (Smith et al., 2004)^7^, AFNI v7.18.1710 (Cox, 1996)^8^, FreeSurfer, v5.1.0 (Dale et al., 1999)^9^, ANTS v.1.9.y (Avants et al., 2008)^10^, ART (as implemented in Nipype)^11^, and PySurfer^12^. Analysis was conducted on a computer cluster running Ubuntu Linux 12.04, 64-bit (kernel 3.2.0-29-generic). Preprocessing and analysis procedures were the same across all TR × ISI permutations, with the exception of specifying the appropriate value for TR when necessary and using the unique stimulus onset times corresponding to each run.

##### Preprocessing

Functional and structural data were converted from Siemens dicom format to nifti using *mri_convert* in FreeSurfer. Cortical reconstruction and parcellation of the anatomical images were performed using the default processing stream in FreeSurfer, the accuracy of which was verified manually via visual inspection.

Correction for participant head motion was achieved by realigning (via six degree of freedom affine transformation) every functional volume to the first functional volume of the first run using the Nipy motion correction algorithm (Roche, 2011). Functional time series did not undergo slice-timing correction due to the discontinuous nature of sparse-sampling. Voxel-wise intensity aberrations in the fMRI time series were reduced using AFNI’s *3dDespike* with default parameters; no other temporal filtering of the functional time series was conducted. To reduce the effects of uncorrelated thermal noise, functional volumes were spatially filtered achieved using FSL’s *SUSAN* algorithm with a 6 mm FWHM 3D Gaussian kernel – an intermediate value chosen to optimize both localization specificity and uncorrelated noise mitigation. Volumes with global intensity differing from the time series mean by more than three standard deviations, or those in which a participant’s composite head motion (the Euclidian combination of head translations and rotations) exceeded 1 mm, were identified by ART and flagged as outliers to be regressed out of the first-level design matrix (one column per outlier).

The coregistration matrix between participants’ mean functional image and their high-resolution anatomy was calculated using FreeSurfer’s *bbregister* with FSL affine initialization and optimization for T2-weighted images. An anatomical mask for constraining the first-level analysis to only intracranial voxels was created using the FreeSurfer aparc+aseg.mgz volume. This volume was binarized, dilated by one voxel, and then transformed from participants’ high-resolution anatomical space to their functional space via FreeSurfer’s *mri_vol2vol* using the inverse of the coregistration matrix calculated by *bbregister* and nearest-neighbor interpolation. The anatomical mask, in native high-resolution space, was used to extract a volume consisting of only brain voxels. This volume was then normalized to the MNI152 1 mm T1 template from FSL via the non-linear symmetric diffeomorphic mapping implemented in ANTS, using the default parameters specified in the *antsIntroduction.sh* script.

##### Model design and estimation

For each participant, for each run, the first-level model was estimated for the preprocessed functional volumes using FSL’s *film_gls*. The design matrix included the task regressor (either the boxcar model of event TAs, or the model incorporating HRF convolution as described in the simulations above (Figures 3 and 4), with a scaled peak-to-peak height of 1.0), as well as the six motion parameters (*x*, *y*, *z* translations; pitch, roll, yaw rotations), the first three Legendre polynomials to account for low-frequency components of the fMRI time series (such as scanner drift), and any nuisance regressors corresponding to outlier volumes identified in ART. The only contrast of interest was task versus rest (implicit baseline).

For each participant, the effect size and variance files for the task vs. rest contrast were merged into 4D files and the second-level, within-participant effect was estimated using FSL’s *flameo* using a weighted fixed-effects model. The resulting second-level effect size and variance files were then transformed into participants’ high-resolution anatomical space via *mri_vol2vol* using the coregistration matrix calculated during preprocessing by *bbregister* and trilinear interpolation. The coregistered files were then normalized via *WarpImageMultiTransform* using the transformation matrix and deformation field between participants’ anatomy and the template space, calculated by ANTS during preprocessing.

##### Combined results across participants

To allow for comparison between the boxcar and HRF model, the pairwise differences between participants’ second-level effect size and *t*-statistic volumes (in normalized space) from each model were calculated using *fslmaths* (i.e., β_HRF_ – β_Box_ and *t*_HRF_ – *t*_Box_). These difference volumes were merged into 4D files, and a mean volume was calculated using *fslmaths*. Group-level statistical parametric (“activation”) maps were computed by estimating an ordinary least squares mixed effects general linear model on participants’ effect size and variance images in normalized space using FSL’s *flameo*. The results of the average pairwise model differences and group-level results were projected to the cortical surface of the MNI template for visualization using FreeSurfer’s *mri_vol2surf* via PySurfer, using intensity values averaged 1mm outward from the white matter surface.

### Results

#### In-scanner behavior

Analysis of in-scanner behavior was conducted in **R** (version 2.15.0); the linear mixed effects model implemented in the “nlme” package was used for statistics with repeated measures. Sensitivity scores (as *d*′) were calculated for each participant in each condition. All participants exhibited very high accuracy during the task (all *d*′ > 2.98, mean *d*′ = 4.13). Sensitivity scores were analyzed using a univariate linear mixed effects model with *TR* and *ISI* as fixed factors and *participant* as a random factor. There was no effect of TR [*F*_1,49_ = 2.96, *p* = 0.092] nor ISI [*F*_1,49_ = 1.56, *p* = 0.217], and no interaction [*F*_1,49_ = 0.34, *p* = 0.562]. Full behavioral data and their summary statistics are available as supplementary material online.

#### Task-evoked response

**Figure 8:**
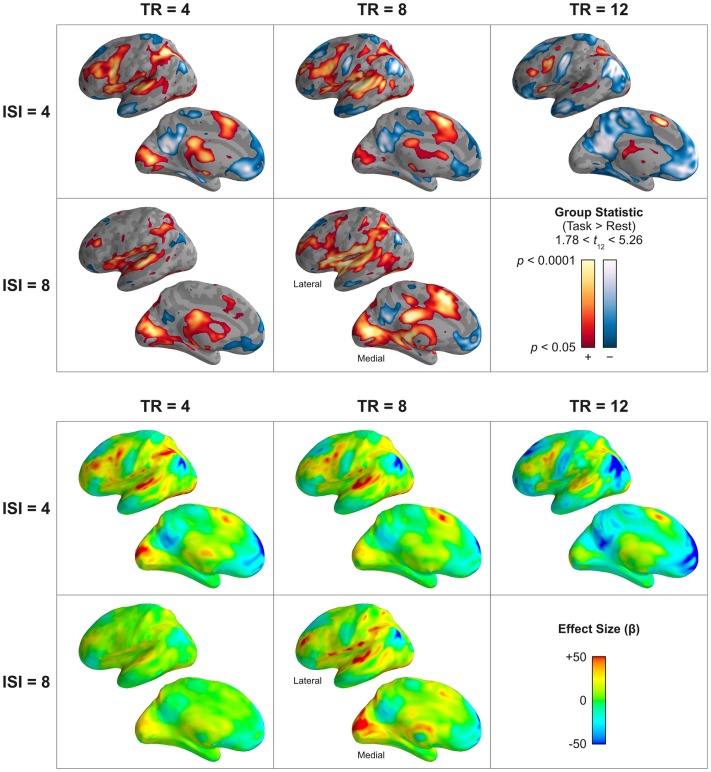
**Group effect of task-related response – as estimated by the HRF model – across stimulation (ISI) and acquisition (TR) rates**. The top panels show the statistical reliability (*t*) and significance (*p*, uncorrected) of the group effect, the bottom panels show the effect size (parameter estimate). A parameter estimate of 50 corresponds to a 5% signal change. The lateral and medial surfaces of the left hemisphere are shown. Shorter acquisition rates and more frequent stimulus presentation tended to produce the most reliable response profile across temporal, frontal, parietal, and occipital cortices. Differences in observed response magnitude in superior temporal cortex between TR = 4 s and TR = 8 s acquisition rates may indicate physiological saturation (reduced stimulus-evoked dynamic range of the BOLD signal) due to differences in the overall amount of acquisition-related scanner noise. However, both the estimated response magnitude and its statistical reliability tended to be dramatically reduced at very long acquisition rates (TR = 12 s) compared to shorter rates (TR = 4 s). Supplementary Figure 1 provides a volume-based depiction (axial slices) of these data. Supplementary Figure 5 illustrates these data for only clusters that survive correction for multiple comparisons.

As designed, the present go/no-go variant of the Stroop task effectively elicited response in an extensive and diverse network of cortical and subcortical regions (Figure 8). The regional pattern of elicited response was largely consistent across variations in TR and ISI, and included the desired suite of auditory (superior temporal gyrus, Heschl’s gyrus), visual (pericalcarine cortex, fusiform gyrus, lingual gyrus), and cognitive (middle frontal gyrus, inferior frontal gyrus (pars opercularis), supplementary motor area, insula, supramarginal gyrus, superior parietal lobe) cortical areas, as well as basal ganglia, thalamus, brainstem, and cerebellum. Regions of deactivation (negative task-related response) were composed of the stereotypical default-mode network, including superior frontal gyrus, anterior middle temporal gyrus, inferior parietal lobe, medial prefrontal cortex, and posterior cingulate.

##### Differences by TR and ISI

All TR × ISI permutations elicited a similar network of brain regions; however, both qualitative and quantitative differences in the magnitude of response were observed. Despite the preponderance of long-delay TR designs in sparse imaging, we observed significantly diminished response in the TR = 12 s design compared to most of the TR = 4 or TR = 8 permutations. For this longest TR, the magnitude of positive response in task-activated sensory and cognitive areas was comparatively small, and the absolute magnitude of response in default-mode areas was comparatively large. The statistical reliability of task-related response in the TR = 12 s design, as determined by the *t*-statistic on the task contrast, was lowest in magnitude and most restricted in volume compared to all other designs.

Unlike the simulations, which showed no systematic difference between the TR = 4 s and TR = 8 s designs in terms of effect size, the results of the actual fMRI experiment demonstrated that the response measured during both TR = 8 s designs was of significantly greater magnitude than the corresponding TR = 4 s design. However, this difference was limited primarily to the superior temporal gyrus and Heschl’s gyrus – areas responsive to auditory stimulation and, therefore, susceptible to saturation from increased acoustic noise as a function of more frequent image volume acquisition during TR = 4 s sampling. This difference in effect size between the TR = 8 s and TR = 4 s designs was reflected in the model reliability: improved model reliability, as measured by the *t*-statistic, was observed for TR = 8 s in auditory regions, indicating that increasing the value of the numerator of the test statistic (the contrast on the parameter estimate) by reducing response contamination from acoustic noise could compensate for larger values in the denominator of the test statistic (greater model error due to smaller sample size). It is worth noting, though, that acoustic noise may not be the only factor affecting differences between TR and ISI permutations. For instance, TR and ISI appear to interact with respect to the effect size observed in visual and superior parietal areas, and response magnitude in the supplementary motor area, despite not being an auditory sensory area, appears to follow the same trend as the STG.

In all cases in the measured fMRI data, more frequent stimulation led to larger responses and more reliable activation in task-responsive areas. For both TR = 4 s and TR = 8 s acquisition rates, designs involving stimulation rates with ISI = 4 s tended to produce larger effect sizes than those with less frequent stimulus events (although in some regions, such as the medial occipital lobe, these design choices appear to have interacted in a more complicated way). These results are largely consistent with the simulations, and demonstrate the efficacy in sparse-sampled fMRI data of the HRF model in capturing the additivity of overlapping hemodynamic responses to repeated stimulation.

##### Differences between HRF and Boxcar models

**Figure 9:**
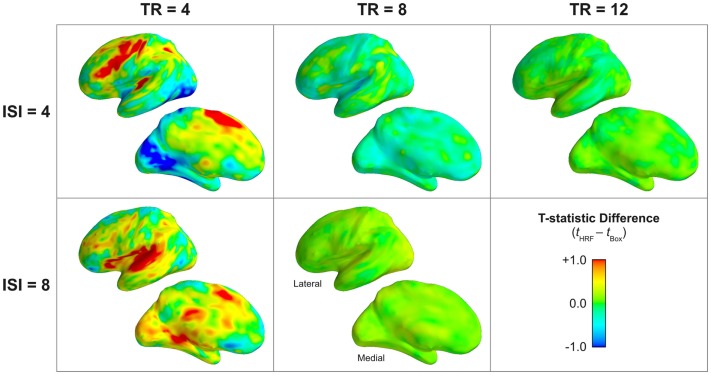
**Increased statistical reliability of sparse models incorporating the hemodynamic response compared to classic boxcar models**. The subject-wise difference between models in the *t*-statistic on the main effect of task is shown, with warm colors indicating increased reliability of the HRF model. The HRF model showed substantial improvement in statistical reliability over the boxcar model in nearly all task-activated cortex. This difference was most prominent for TR = 4 s, but smaller differences were still evident in temporal, parietal, and frontal regions even for TR = 8 and 12 s. The lateral and medial surfaces of the left hemisphere are shown. Supplementary Figure 2 provides a volume-based depiction (axial slices) of these data.

The differences in the statistical reliability of the classical boxcar model versus the physiologically informed model observed in the actual fMRI data (Figure 9) largely corroborated the results of the simulations (Figure 5). For short TR lengths (here, TR = 4 s), the HRF model afforded substantially increased statistical reliability (Δ*t* > 0.7; corresponding to an order of magnitude improvement in statistical significance) across a wide range of task-responsive regions, including, notably, dorsolateral prefrontal cortex, supplementary motor area, auditory cortex, superior temporal gyrus, and the superior parietal lobe. These effects were observed for both faster (ISI = 4 s) and slower (ISI = 8 s) stimulation rates. For longer TR lengths (TR ≥ 8 s), no compelling pattern of whole-brain differences between the models was observed; however, some circumscribed regions did exhibit somewhat modest improvement with HRF modeling.

**Figure 10:**
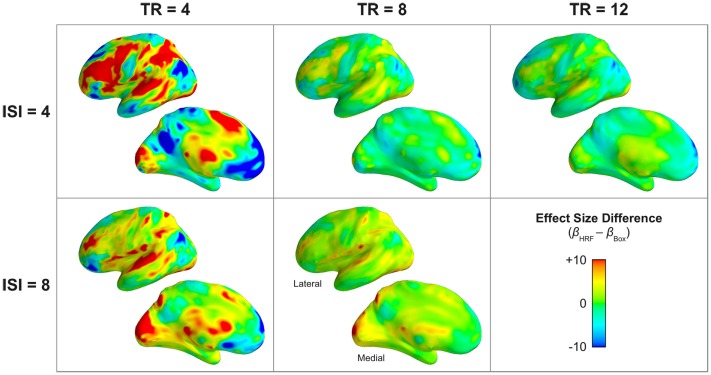
**Classical boxcar models estimate consistently and substantially reduced magnitude of the physiological effect size across acquisition (TR) and stimulation (ISI) rates**. The subject-wise difference between models in the main effect of task is shown. Warm colors indicate regions where the effect size was larger in the HRF model; cool colors indicate larger (more positive) effect sizes in the boxcar model. Note that many of the regions exhibiting “larger” effect sizes under the boxcar model are, in fact, areas corresponding to the default mode network with corresponding task-related deactivation (Figure 7). Thus, for medial prefrontal, posterior cingulate, inferior parietal, superior frontal, and temporal pole, the HRF model estimated larger magnitude “deactivations” than the boxcar model. A parameter estimate of 10 corresponds to a 1% signal change. The lateral and medial surfaces of the left hemisphere are shown. Supplementary Figure 3 provides a volume-based depiction (axial slices) of these data.

Correspondingly, the actual fMRI data validated the results of the simulation with respect to estimated effect size. The boxcar model produced substantially lower estimates of the magnitude of physiological percent signal change compared to the HRF model, as determined by the difference between models’ parameter estimates for the effect of task (Figure 10). In task-activated frontal, temporal, parietal, and occipital regions, the boxcar model produced lower effect size estimates by up to 1 percentage point for long TRs (TR ≥ 8 s), and by up to 5 percentage points for the shortest measured TR (4 s). In addition to lower estimates of the magnitude of task-related activation, the boxcar model estimated smaller task-related deactivations, as well. In Figure 10, areas of apparently greater effect size under the boxcar model (e.g., medial prefrontal cortex, posterior cingulate, inferior parietal, etc.) correspond exactly to those default-mode network regions exhibiting task-related deactivations; that is, the HRF model estimated negative responses of greater absolute magnitude than the boxcar model did in these regions.

#### Disentanglement of “go” and “no-go” conditions in the HRF model

A major limitation of the classic boxcar model approach to sparse analysis is its inherent inability to distinguish between multiple event types presented or elicited during a single TR delay. As such, this has effectively limited the types of fMRI experimental paradigms available in sparse scanning to either block-designs or slow event-related designs. Even if a rapid event-related design were attempted using a boxcar model and short TR, its efficiency would be compromised because of overlapping hemodynamic responses to different event types sampled in a single TA (Figure 2). A major advantage of the use of a physiologically informed model incorporating convolution of the canonical HRF in sparse imaging is that it allows for the disentanglement of the overlapping hemodynamic responses of different event types (following Dale, 1999), thereby making available experimental designs (for any TR delay length) in which stimuli of different event types can be presented during a single TR delay.

We assessed the efficacy of this approach by repeating the within-participant and group-level model design and estimation described in the fMRI methods above, but this time including two separate task regressors for the “go” (matching text color/spoken color name) and “no-go” trials, respectively (Figure 7). The contrast between these parameters was estimated in the within-participant first-level analysis, and the contrast parameter estimates and variance volumes were carried forward through the second-level fixed-effects and group-level mixed effects models described above.

**Figure 11:**
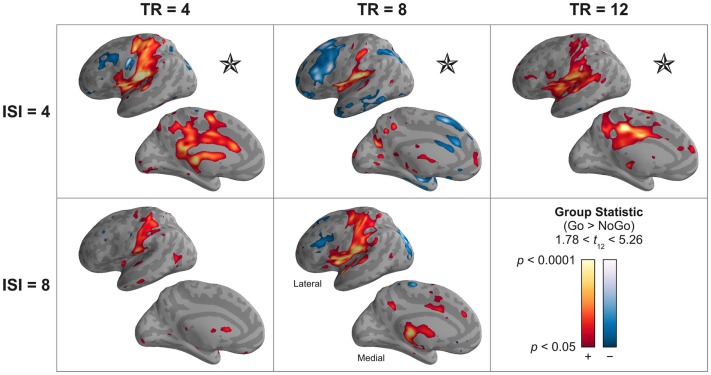
**A sparse model incorporating convolution of the canonical HRF can distinguish among multiple event types occurring within a single silent delay**. The statistical reliability (*t*) and significance (*p*, uncorrected) of the group effect of the contrast “Go” > “No-Go” events is shown. Warm colors correspond to greater activation during “Go” events; cool colors, “No-Go” events. Stars (⋆) indicate those designs in which the hemodynamic responses to different event types overlap during a single image volume acquisition (TA), demonstrating the ability of the HRF model to reliably and correctly estimate differential response to these events. Minor variations in the pattern of activation across designs may be the result of differences in the relative efficiency of the presentation order of “Go” and “No-Go” events rather than specific effects of the TR or ISI. The lateral and medial surfaces of the left hemisphere are shown. Supplementary Figure 4 provides a volume-based depiction (axial slices) of these data. Supplementary Figure 6 illustrates these data for only clusters that survive correction for multiple comparisons.

In this analysis, regions showing a differential Go > No-Go response included somatomotor cortex, insula, and posterior cingulate; a No-Go > Go response was observed in dorsolateral prefrontal cortex and superior parietal lobe. This network of regions was highly consistent across permutations of TR and ISI length (Figure 11). Importantly, these regions could be reliably discerned for both designs in which only a single event type contributed to the hemodynamic response sampled by each TA (both ISI = 8 s designs), and those in which the two event types both contributed to the hemodynamic response sampled during a single TA (all ISI = 4 s designs).

#### Open access dataset

The original data comprising this report are available online via the MIT institutional repository DSpace at http://dspace.mit.edu/handle/1721.1/68161. Files available for download include, for each TR × ISI permutation and analysis model: (1) individual subjects’ analyzed fMRI data (*t*-statistics, variance, and effect size maps) in normalized space, (2) group-level data (*t*-statistics and effect size maps), and (3) fMRI analysis scripts.

### Discussion

The results of the fMRI experiment largely validated the results of the computational simulations, while extending them by revealing a number of interactions between experiment design and the nature of the MR scanning environment that were not included in the simulations. First, we again observed the benefit to effect size afforded by more frequent stimulus presentation. Second, we also observed increased model reliability for more frequent image volume acquisition (TR = 4, 8 s) than for longer TRs (12 s). This difference in statistical reliability can be explained in part by the larger number of sample volumes over which the model is estimated, as seen in the simulations; however, the significantly smaller parameter estimate for task activation in the TR = 12 s design was not observed in the simulations. This suggests that sparse models are less effectively estimated for the long TR design, or that other psychophysiological interactions, brought about by long delays between scanner acquisitions, may have contributed to a reduction in task-related response. Correspondingly, the disproportionately large magnitude of default-mode network deactivation at TR = 12 s compared to the other conditions was somewhat surprising. Although it has been shown that the default-mode network is more reliably evoked during sparse than continuous sampling (Gaab et al., 2008; Langers and van Dijk, 2011), the effects of varying sparse delay in these regions has not previously been examined. Although only speculation, it may be the case that, because we constrained rest events to last the full length of a TR, long periods of rest like those seen in the TR = 12 s condition might be more efficacious at eliciting the default-mode network than the briefer rest periods of the shorter TR delays.

Unlike the simulations, in the actual fMRI data we observed a difference in estimated response magnitude in auditory areas between TR = 4 s and TR = 8 s designs, with larger effect sizes favoring less frequent image volume acquisition. This is likely the result of increased auditory response contamination due to more frequent acoustic noise associated with shorter TRs: response in auditory cortex begins to saturate as a result of scanner gradient noise during EPI acquisition, reducing the dynamic range available for observing task-elicited response (Langers et al., 2004; Scarff et al., 2004). This result suggests that, for TRs of an intermediate delay, there exists a trade-off between reducing the denominator of the test statistic through shorter TRs that reduce model error, and increasing the numerator of the test statistic through longer TRs that permit auditory responses of larger magnitude. Although we did not have the opportunity to investigate further parameterization of the TR delay in this study, based on the results of our simulations (Figure 6), and other studies utilizing sparse design being conducted by our group, we suggest that the “sweet spot” for maximizing the combined benefits of longer TR delay and more frequent sampling might be in the vicinity of TR = 6 s. Although one might expect the regions primarily affected by this trade-off to be primary and association auditory cortex, whereas other, non-auditory regions would not be sensitive to the effects of acoustic noise, the patterns of activation observed in the whole-brain (Figure 8) suggest there might be a more complicated relationship between sampling frequency and physiological response magnitude across regions.

Finally, and perhaps most importantly, the actual fMRI results validated the substantial improvements to model reliability and effect size estimation afforded by a physiologically informed model incorporating the canonical HRF. As in the simulations, this result was most apparent for short TRs. Moreover, the use of the HRF model allowed for the reliable estimation of different event types that co-occur in a single TR, and whose hemodynamic responses overlap during a single TA. In the present experiment, this allowed us to distinguish between “go” and “no-go” trials, but one can easily envision the benefits afforded by application of this technique to more scientifically interesting questions in auditory cognitive neuroscience, such as voice selectivity; mapping cortical representations of articulators; tonotopic mapping, etc.

## Conclusion

Through computational simulations and validation with actual fMRI data, we explored the parameter space of sparse-sampling fMRI design and analysis. Specifically, we investigated the effects and interactions among the length of silent delay during the TR (rate of image volume acquisition) and ISI (rate of stimulus presentation) as design parameters, and the use of classical boxcar or physiologically informed models incorporating HRF convolution as an analysis parameter. Based on a synthesis of these results, we suggest that, in many (but certainly not all) situations, the following methods may be employed to help optimize signal detection in sparse-sampling fMRI experiments:

1. Utilize a model incorporating canonical hemodynamic response convolution (Figure 2). This will generally afford improved statistical significance (Figures 5 and 9), resulting in part from more accurate estimation of physiological response magnitude (percent signal change) (Figures 5 and 10). Moreover, sparse models that account for the hemodynamic response enable the use of rapid event-related sparse designs that can distinguish the effects of different event types whose hemodynamic responses overlap during a single volume acquisition (Figures 3D and 11). Note, however, that care needs to be taken when there is reason to believe the canonical hemodynamic response function may not accurately correspond to the true physiological response (Aguirre et al., 1998; Harms and Melcher, 2003).
2. Maintain a high rate of stimulus presentation (Figures 6 and 8). Presenting stimuli more frequently, when combined with an HRF-convolved model, takes advantage of hemodynamic response additivity and increases measured response magnitude. Although this approach suggests a departure from slow event-related sparse designs (Belin et al., 1999), such designs – although inherently less efficient (Dale, 1999) – will still serve effectively in certain situations, such as determining the shape of auditory hemodynamic responses.
3. Acquire image volumes after a short or intermediate TR delay (Figures 6 and 8). Whereas the classic approach in sparse design has been to utilize very long TRs (>12 s) to completely avoid acoustic contamination of the measured response, all the present data suggest that such an approach is sub-optimal. Shorter TR delays (2–4 s) allow for the collection of a larger number of image volumes, which reduces model error and improves both statistical reliability and significance. Intermediate TR delays (4–6 s) may compensate for reduced model power by reducing acoustic contamination and increasing the measurable effect of auditory stimulation – a benefit that may be limited to primary and association auditory cortices. However, it is important to note that in order to measure physiological responses to auditory stimuli completely devoid of contamination by response to the acoustic noise of scanner acquisition, very long TRs (>16 s) will still be required.

We hope these results serve as guidelines for the development of more effective sparse-sampling fMRI experiments and facilitate the effective application of these techniques in pursuit of discoveries in auditory cognitive and systems neuroscience. It is worth pointing out that other technological or statistical advances may be added to these optimizations to further enhance the reliability of fMRI experimentation. For example, it is reasonable that the HRF model could be further improved by the inclusion of additional parameters in the design matrix modeling the temporal and spread derivatives of the hemodynamic response, reducing the cost of variability in HRF across participants. Likewise, advances in quieter acquisition sequences or better acoustic isolation may also reduce the effect of acoustic contamination on the dynamic range of auditory cortex, allowing further benefit to be derived from shorter (versus intermediate) TRs. In addition to these data, we have made the simulation code available. Prior to beginning a new sparse experiment, researchers may find it advantageous to utilize computational models simulating various experiment designs – including acquisition rates, stimulus jitter with respect to the TA, etc. – to determine a design that will optimize the detection of an effect or contrast of interest. It should be noted, however, that such simulations cannot capture complex interactions between the acoustic noise of scanner acquisition and the reduced dynamic range of physiological response in the auditory cortex.

Finally, we end with an appeal to the community of neuroimagers using sparse-sampling to be as descriptive as possible in reporting model design and analysis parameters. At a minimum, methods sections describing sparse imaging should indicate the TA, delay length, stimulus jitter with respect to the TA, number of volumes acquired during each acquisition, what basis functions were used in the model, how discontinuity in the sparse time series was handled in the model, whether any temporal filtering or correction for temporal autocorrelation was done and how, how the task regressors in the model were scaled, and, if percent signal change is reported, how it was calculated. These, in addition to the standard parameters expected in methods sections (Poldrack et al., 2006; Carp, 2012) should improve the replicability and generalizability of sparse-sampling fMRI results.

## Conflict of Interest Statement

The authors declare that the research was conducted in the absence of any commercial or financial relationships that could be construed as a potential conflict of interest.

## Supplementary Material

The Supplementary Material for this article can be found online at http://www.frontiersin.org/Brain_Imaging_Methods/10.3389/fnins.2013.00055/abstract

## Footnotes

^1^In our view, the term “sparse-sampling” should be used to describe any acquisition paradigm involving a silent delay between functional volume acquisitions and should mean, by implication, that a single functional volume was collected for each delay. The modifier “clustered” should, in contemporary and future usage, be reserved for acquisition paradigms involving the collection of multiple contiguous functional volumes between silent delays. We maintain this distinction henceforth in this report.

^2^**R**: http://www.r-project.org/

^3^The principal real-world constraint on the amount of fMRI data available for any particular experiment is the limited availability of MRI scanner time, both because of its substantial expense for an individual investigator and because of its high demand within a shared imaging center. As such, in our simulations we sought to approximate real-world design considerations by limiting our analysis to runs of a consistent and constrained length across variations in parameters. We selected a constant run duration of 360 s, both because this is typical of real-world experiments and, importantly, because the large number of divisors of this number allowed the simulation of a wide variety of realistic TR lengths.

^4^Praat: http://www.fon.hum.uva.nl/praat/

^5^NiPype: http://nipy.sourceforge.net/nipype/

^6^BIPs: https://github.com/INCF/BrainImagingPipelines

^7^FSL: http://fsl.fmrib.ox.ac.uk/fsl/fslwiki/

^8^AFNI: http://afni.nimh.nih.gov/afni/

^9^FreeSurfer: http://surfer.nmr.mgh.harvard.edu/

^10^ANTS: http://www.picsl.upenn.edu/ANTS/

^11^ART: http://www.nitrc.org/projects/artifact_detect/

^12^PySurfer: http://pysurfer.github.com/
